# System-Level Insights into Yeast Metabolism by Thermodynamic Analysis of Elementary Flux Modes

**DOI:** 10.1371/journal.pcbi.1002415

**Published:** 2012-03-01

**Authors:** Stefan J. Jol, Anne Kümmel, Marco Terzer, Jörg Stelling, Matthias Heinemann

**Affiliations:** 1 Life Science Zurich PhD Program on Systems Biology of Complex Diseases, ETH Zurich, Zurich, Switzerland; 2 Institute of Molecular Systems Biology, ETH Zurich, Zurich, Switzerland; 3 Department of Biosystems Science and Engineering, ETH Zurich, Basel, Switzerland; 4 Swiss Institute of Bioinformatics, ETH Zurich, Basel, Switzerland; 5 Molecular Systems Biology, Groningen Biomolecular Sciences and Biotechnology Institute, University of Groningen, AG Groningen, The Netherlands; University of Virginia, United States of America

## Abstract

One of the most obvious phenotypes of a cell is its metabolic activity, which is defined by the fluxes in the metabolic network. Although experimental methods to determine intracellular fluxes are well established, only a limited number of fluxes can be resolved. Especially in eukaryotes such as yeast, compartmentalization and the existence of many parallel routes render exact flux analysis impossible using current methods. To gain more insight into the metabolic operation of *S. cerevisiae* we developed a new computational approach where we characterize the flux solution space by determining elementary flux modes (EFMs) that are subsequently classified as thermodynamically feasible or infeasible on the basis of experimental metabolome data. This allows us to provably rule out the contribution of certain EFMs to the in vivo flux distribution. From the 71 million EFMs in a medium size metabolic network of *S. cerevisiae*, we classified 54% as thermodynamically feasible. By comparing the thermodynamically feasible and infeasible EFMs, we could identify reaction combinations that span the cytosol and mitochondrion and, as a system, cannot operate under the investigated glucose batch conditions. Besides conclusions on single reactions, we found that thermodynamic constraints prevent the import of redox cofactor equivalents into the mitochondrion due to limits on compartmental cofactor concentrations. Our novel approach of incorporating quantitative metabolite concentrations into the analysis of the space of all stoichiometrically feasible flux distributions allows generating new insights into the system-level operation of the intracellular fluxes without making assumptions on metabolic objectives of the cell.

## Author Summary

Fluxes in metabolic pathways are a highly informative aspect of an organism's phenotype. The experimental determination of such fluxes is well established and has proven very useful. To address some of the limitations of experimental flux analysis, such as when the cell is divided in multiple compartments, stoichiometric modeling provides a valuable addition. The approach that we take is based on stoichiometric modeling where we consider the thermodynamic feasibility of many different possible routes through the metabolic network of *Saccharomyces cerevisiae* using experimentally determined metabolite concentrations. We show that next to conclusions on single biochemical reactions in the metabolic network, we obtain system-level insights on thermodynamically infeasible flux patterns. We found that the compartmental concentrations of  and NADH are the causes for the system-level infeasibilities. With the current advances in quantitative metabolomics and biochemical thermodynamics, we envision that the presented method will help gaining more insight into complex metabolic systems.

## Introduction

Metabolic fluxes give immediate insights into the metabolism of a cell [1], [2]. Metabolic flux analysis has proven to be useful, for example for the determination of enzyme functions [3], for the identification of regulatory mechanisms in response to environmental perturbations [4], or as a tool in metabolic engineering [5]. The most common method to quantify metabolic fluxes uses 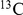 labeled substrates, and the measured label distribution in intracellular metabolites is interpreted together with measured uptake and production rates by means of a metabolic network model [6].

Despite successful quantification of fluxes with 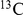 flux analysis in different conditions, the method has several limitations. For example, it is limited to the main branches in central carbon metabolism and fluxes cannot be resolved per compartment [7], despite compartmentation being a highly relevant aspect of eukaryotes [8]. Moreover, today's 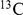 flux analysis rests on a number of a priori assumptions, e.g. on reaction reversibilities or on relevant parts of the network [7], [9].

To improve flux quantification, we need additional constraints on the possible flux distributions in a metabolic network. In stoichiometric network analysis, the metabolic network is modeled as a collection of biochemical reactions where all internal metabolite concentrations are assumed constant [10]. Next to the typical constraints, such as uptake and excretion rates, reaction reversibilities and maximum flux capacities, the field recently began to incorporate thermodynamic information, whereby statements on feasibility of reaction fluxes or flux distributions can be made based on calculation of changes in Gibbs energy using metabolite concentrations [11]–[13]. For example, using flux balance analysis (FBA) and related approaches, metabolite concentrations were used as additional constraints to predict fluxes in the non-compartmentalized organism *E. coli* [14], [15] or in a model of liver metabolism [16].

Here, we develop a novel approach to integrate metabolite data into metabolic network flux analysis, to get additional insight into the compartmentalized flux physiology of *Saccharomyces cerevisiae*. The method combines network embedded thermodynamic (NET) analysis [12], elementary flux mode (EFM) analysis [17]–[19], and experimentally determined metabolome data. We employ EFM analysis instead of flux balance analysis because the collection of the generated flux modes can yield insight into all feasible flux distributions, as compared to the single thermodynamically feasible flux solution that is obtained with thermodynamically constrained FBA. Additionally, assumptions on a metabolic objective function of the cell are not required.

Our new approach to analyze the compartmentalized central metabolic network of *S. cerevisiae* using quantitative metabolite data acquired under glucose batch growth conditions allowed us to generate novel insight into the system-level causalities underlying the intercompartmental redox metabolism. Specifically we show that the 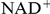 and NADH concentrations in the cytosol and the mitochondrion do not allow for the ethanol-acetaldehyde redox shuttle to be active under the investigated condition. Further, we identified a number of maximal reaction activities that could be used as constraints for 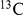 flux analysis or FBA. We envision that our method becomes a useful tool to unravel system-level insights about a complex metabolic system from metabolome data.

## Results

Our approach uses elementary flux modes (EFMs) to describe metabolic flux distributions. The concept of EFMs is well known for stoichiometric network analysis, and it provides a way to explore the flux solution space of a metabolic network that is commonly addressed with flux balance analysis (FBA). With all the EFMs of a metabolic network, any stoichiometrically possible flux distribution can be obtained by a non-negative linear combination of the EFMs [20]. Since we want to evaluate only thermodynamically feasible flux distributions, we demonstrate, as a first step towards the development of our approach, a new property of EFMs, which is that every thermodynamically feasible flux distribution is a non-negative linear combination of thermodynamically feasible EFMs.

### All thermodynamically feasible flux distributions can be generated by the set of thermodynamically feasible EFMs

The mass balanced flux solution space of a stoichiometric metabolic network can be described with a non-negative linear combination of its EFMs:
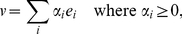
(1)where any flux distribution 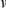 is a sum of EFMs 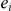 with coefficients 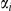.

As we show in the proof provided in Text S1, a thermodynamically feasible flux distribution only consists of thermodynamically feasible EFMs:
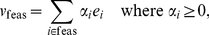
(2)where the thermodynamically feasible flux distribution 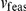 is only composed of EFMs from the feasible set, 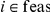.

The mathematical proof demonstrates that by eliminating infeasible EFMs we do not loose feasible flux distributions, because any feasible flux distribution can be composed of only feasible EFMs. Specifically, in the hypothetical case that an infeasible EFM is part of a feasible flux distribution it must involve a cancellation or directionality change of a specific reversible reaction. In this case, the flux distribution can be decomposed into one or more feasible EFMs, and a feasible combination of an infeasible EFM with another EFM. The combination of the infeasible EFM and another EFM must then be either a feasible EFM by itself, or it must be possible to achieve the feasible combination by other feasible EFMs. Hence, Eq. (2) allows us to exclude thermodynamically infeasible EFMs from the complete set of EFMs without excluding thermodynamically feasible flux distributions. It is important to note that the resulting flux solution space defined by the feasible EFMs can still contain infeasible flux solutions because it is possible that the combination of multiple feasible EFMs leads to an infeasible flux distribution.

### From network stoichiometry to EFMs and thermodynamic classification

Exploiting that EFMs allow us to exclude thermodynamically infeasible EFMs, we aimed at developing an approach to generate novel insights into the complex flux physiology of the central metabolism of the yeast *S. cerevisiae*. Therefore, we assembled a 230 reaction stoichiometric network of its central carbon metabolism and amino acid synthesis pathways (cf. Materials and Methods) encompassing the cytosolic and mitochondrial compartments and many parallel pathways. With our approach we aim to obtain additional insight into the metabolic network operation, therefore we build upon current knowledge by defining the reversibilities in our model as they are defined in the original model [21].

**Figure 1:**
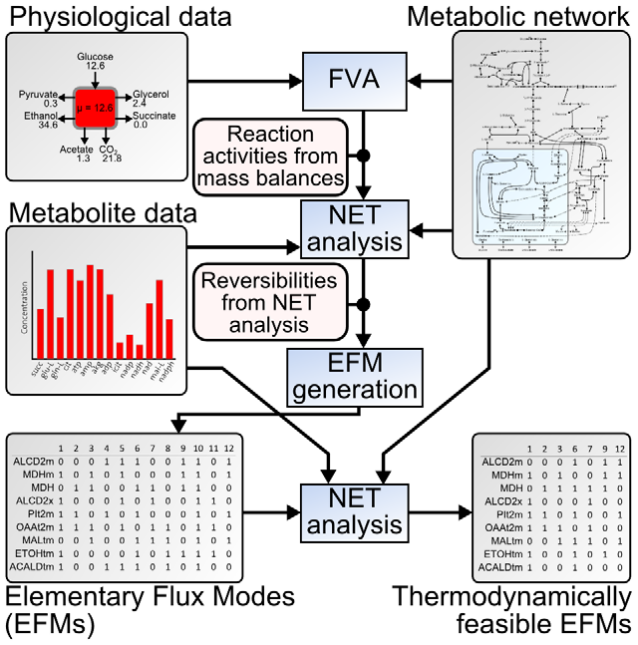
Overview of procedure to identify thermodynamically feasible EFMs. We use a metabolic network model of *S. cerevisiae* together with physiological data (e.g., growth rate, glucose uptake, ethanol production) in flux variability analysis (FVA) to determine reaction reversibilities and reaction activities for the specific conditions. Subsequently, we apply NET analysis to determine additional reaction direction constraints based on the activities of reactions that are active for all flux distributions. With the resulting condition specific reaction directionalities, we calculate all the EFMs for the metabolic network. Finally, we use quantitative metabolite data to test the reaction activities of each EFM for thermodynamic feasibility.

**Figure 2:**
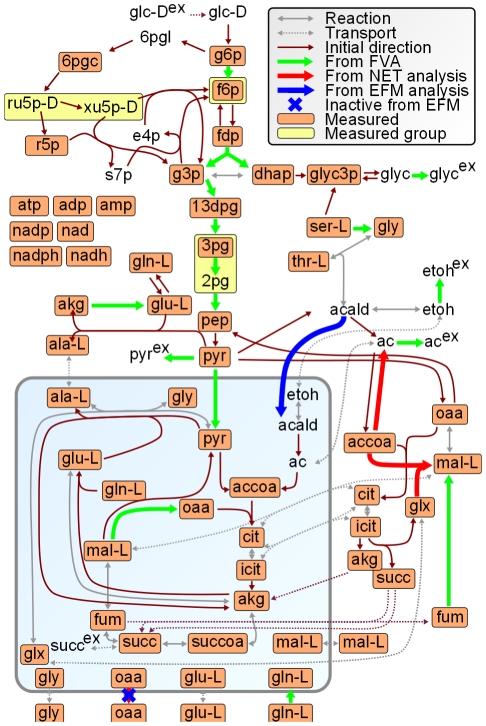
Overview of the main reactions in the metabolic model of *S. cerevisiae*. The shaded area indicates the mitochondrion. The colors of the directed arrows indicate from which information a constraint was added. The blue cross indicates the oxaloacetate transported that is predicted to be inactive after analyzing the thermodynamically feasible EFMs. For a list of metabolite names see Dataset S2.

First, we needed to calculate all EFMs. As the EFM calculation is computationally demanding, we initially applied steps to constrain the mass-balanced solution space as much as possible upfront, before we started enumerating EFMs (Fig. 1). Thus, in a first step, we performed flux variability analysis (FVA) [22] on the basis of the measured uptake and production rates of external compounds and biomass, to determine the reversibility of each reaction under the investigated physiological conditions, that is, for growth on glucose. From FVA, we obtain a minimum and maximum achievable flux for each reaction. A reaction is reversible if both a negative and positive flux can be achieved, else it is unidirectional. Reactions that have a minimum and maximum flux that are either positive or negative, and cannot be inactive, are reactions that are always active in the respective direction. Using this approach, we could classify 67 initially reversible reactions as unidirectional (Fig. 2, Dataset S1).

Next, we employed measured metabolite concentrations from glucose batch cultures, and NET analysis to identify additional reaction irreversibilities [12], [23]. For the metabolite data, we assembled published and unpublished data from glucose batch experiments, and generated a consensus data set to define lower and upper concentration limits for 55 metabolites (see Materials and Methods and Dataset S2). For NET analysis, we used the reaction activities inferred from FVA. With the consensus metabolite data set, we obtained constraints on the reversibility of three additional reactions (see Fig. 2). Another iteration of FVA and NET analysis with the obtained constraints as input did not yield any further constraints.

For the obtained condition-specific constrained metabolic network, we computed the EFMs and obtained 71.266.960 EFMs. Using NET analysis and the consensus metabolite data set, we classified 38.420.207 (54%) EFMs as feasible and 32.846.753 (46%) EFMs as infeasible. Assuming that the flux solution space in the metabolic network of an organism can be approximated by the number of EFMs, our result shows that roughly at most half of the solution space is thermodynamically feasible.

### From EFMs to flux insight

With the finding that 54% of all the EFMs are thermodynamically feasible we reduced the number of EFMs that can constitute a thermodynamically feasible flux distribution considerably. In a first analysis step towards generating insights into the flux distribution, we searched the feasible EFM set for reactions that only use a subset of the possible reaction directions compared to the complete set of EFMs. For each reaction in each EFM we determined whether a backward, inactive or forward reaction activity was used. Then, the possible reaction activities for each reaction were compared between the complete set of EFMs and the feasible set of EFMs.

Here, we found that the oxaloacetate transport from the mitochondrion to the cytosol is never used in an EFM of the feasible set, meaning that it has to be inactive under the investigated growth condition. Indeed, we find no contradicting evidence for the prediction when comparing with the experimental observation that a knock-out of the corresponding gene OAC1, whose translated protein is responsible for the respective oxaloacetate transport reaction, does not have an effect on growth rate under glucose batch conditions [24], [25]. Further, as we found that all EFMs with acetaldehyde transport out of the mitochondrion are infeasible, we conclude that during growth on glucose, acetaldehyde can only be transported *into* the mitochondrion. It is important to note that the EFMs with active oxaloacetate transport, or acetaldehyde transport out of the mitochondrion, are not infeasible because of the metabolite concentration constraints on the respective single reaction only, since single reaction infeasibilities are removed in the first NET analysis step before EFM generation (see Fig. 1). Instead, as we will show later, the infeasibility is the result of a system of coupled reaction activities, where all individual reactions need to be thermodynamically feasible simultaneously.

**Figure 3:**
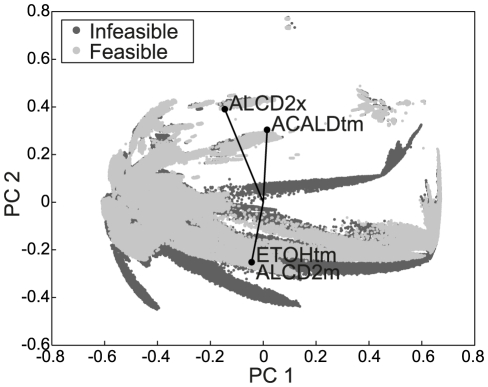
Principal component analysis on normalized EFMs represented in a matrix with each EFM as a sample and each reaction as a variable. All reaction rates are normalized to the glucose uptake rate of the respective EFM. The projection of the data (dots) for principal components one and two (PC 1 and PC 2), and the coefficients of the highest loadings in component 2 (labeled vectors) are shown. The reactions with highest loadings in PC 2 are likely to be involved in the causes for infeasibility of EFMs. One million EFMs were sampled to perform the PCA, multiple repeats with random sampling gave similar results.

Next, we wanted to test whether the feasible set of EFMs differs from the infeasible set in terms of reaction rates. Such a comparison is possible by normalizing the reaction rates in each EFM to the glucose uptake rate of the EFM (all EFMs have glucose uptake, EFMs without glucose uptake are internal cycles and they were removed because they are physiologically meaningless [26]). Principal component analysis (PCA) of the normalized EFMs shows a clear difference between the feasible and infeasible EFMs in principal component 2 (PC 2 in Fig. 3). The reactions with the highest loadings in this component are alcohol dehydrogenase in both the cytosol and the mitochondrion (ALCD2x, ALCD2m) and acetaldehyde and ethanol transport to the mitochondrion (ACALDtm, ETOHtm), and these reactions are likely involved in causing thermodynamic infeasibilities. Note, that although there is a separation between feasible and infeasible EFMs because there are no feasible EFMs in the center of the graph, by combination of feasible EFMs it could still be possible to obtain a feasible flux distribution that would be projected in this area of the PCA. Therefore, the loadings of PC1 are not considered.

**Figure 4:**
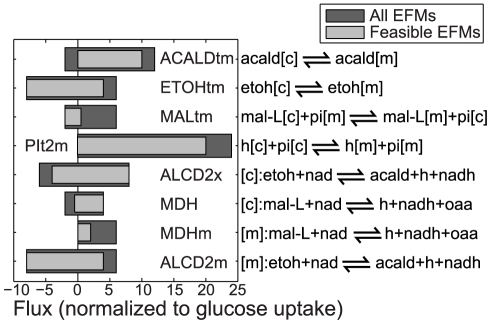
Ranges of possible flux values for eight reactions for which a reduction in the feasible flux range was found, during growth on glucose. The dark shaded bars show the possible flux range obtained from all EFMs, while the light shaded bars show the ranges that are obtained from the feasible EFMs. In the reaction formulas the [c] and [m] indicate that the metabolite or overall reaction occur in the cytosol or mitochondrion, respectively. Abbreviations: acald (acetaldehyde), etoh (ethanol), mal-L (malic acid), pi (phosphate), h (proton), nad (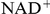), nadh (NADH), oaa (oxaloacetic acid).

Next, we searched for the highest and lowest rate of each reaction in the complete set of EFMs and in the feasible set of EFMs to define the flux ranges that can be achieved in terms of flux per unit of glucose uptake. Any flux value in this range can in principle be achieved through a combination of EFMs. When comparing the flux ranges that can be realized by the feasible EFMs with the flux ranges of the complete set of EFMs, we find that eight reactions cannot assume the full range for thermodynamic reasons (see Fig. 4), with four of these reactions already having shown high loadings in the second principal component (see Fig. 3). These quantitative flux constraints result from metabolite concentrations and thermodynamics, and can be applied as constraints in flux balance analysis.

#### Causes for infeasibility

Next, we aimed to identify the causes for the infeasibility of EFMs. In general, the infeasibility of an EFM is caused by a combination of multiple reaction activities that conflict with the metabolite concentrations due to thermodynamics. We refer to these infeasible combinations of reaction activities as patterns. It is interesting to determine such patterns as they subsequently allow biological interpretation.

**Figure 5:**
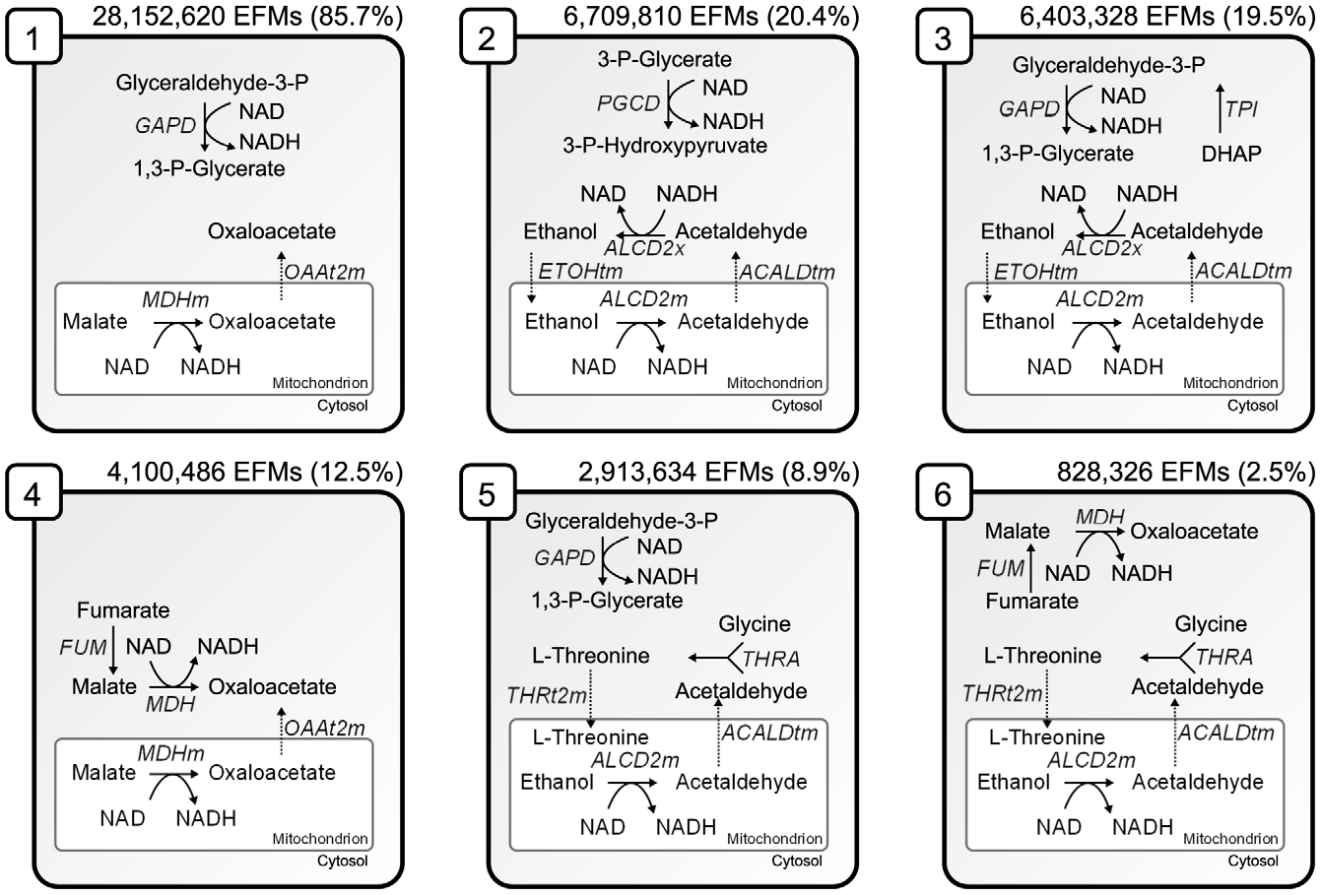
Overview of the six reaction patterns that cause thermodynamic infeasibility of EFMs. The number above each pattern indicates in how many EFMs the respective pattern occurs, and the number in bracket shows the percentage of the infeasible EFMs that contains the pattern.

All identified six patterns involve one of the two earlier mentioned transport reactions (mitochondrial oxaloacetate and acetaldehyde transport) (see Fig. 5). The inactivity of oxaloacetate transport is due to the infeasibility of patterns 1 and 4. In pattern 2 and 3, we find that a part of the pattern is the ethanol-acetaldehyde redox shuttle, which “transports” NADH from the cytoplasm into the mitochondrion. This means that under the investigated glucose batch conditions, a flux distribution with an active ethanol-acetaldehyde redox shuttle is thermodynamically infeasible when operating together with either triosephosphate isomerase (TPI) and glyceraldehyde-3-phosphate dehydrogenase (GAPD) or phosphoglycerate dehydrogenase (PGCD) in the directions as we expect them to occur during growth on glucose as the sole carbon source (Fig. 5). The ethanol-acetaldehyde redox shuttle itself (i.e., only the combination of the activities of the mitochondrial and cytosolic alcohol dehydrogenases and the transporters) is feasible. However, it always occurs in EFMs in combination with the activities of either the TPI and GAPD or PGCD reactions, which makes the EFM as a system infeasible. The biological meaning of the system-level constraints of patterns 2 and 3 corresponds well with what is known about yeast metabolism in glucose batch growth where glucose is mainly converted to ethanol. Thus, the NADH produced in the conversion of glyceraldehyde-3-phosphate to 1,3-biphosphoglycerate, is re-oxidized again to 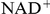 in the conversion of acetaldehyde to ethanol [27].

The identified patterns can also be used as possible constraints in FBA approaches. To confirm whether using such a constraint would be of value, we checked for the presence of the patterns in FBA predictions. Interestingly, it turns out that the identified infeasible flux patterns do not occur in FBA-predicted flux distributions using the objective function “maximization of the ratio of ATP production over the sum of squared fluxes” that was identified to be suitable for glucose batch conditions [28]. In contrast, FBA solutions obtained with the objective functions maximization of ATP production [29], which was found to be suitable for glucose growth in a chemostat, and maximization of biomass production do contradict the patterns (see Text S2).

#### Thermodynamic constraints in redox metabolism

Strikingly, all the patterns in Fig. 5 involve NAD(H) consuming/producing reactions in both the cytosol and the mitochondrion, which may point to infeasibilities in redox reactions. Therefore, we can ask, what makes the patterns in Fig. 5, and specifically the ethanol-acetaldehyde redox shuttle, infeasible, and what effect does this have on redox metabolism?

To address this question, we analyzed the individual metabolite concentration constraints by releasing one concentration constraint at a time. We found that the concentrations of 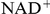 and NADH in both the cytosol and the mitochondrion cannot have values that agree with both the metabolite data and the directionality constraints. For example, for pattern 3 to satisfy the reaction direction constraints the required NADH concentration in the cytosol needs to be low (between 0.1 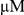 and 0.13 mM), while according to the concentration data constraints the concentration needs to be between 0.23 mM and 1.6 mM. It is important to note that the constraints on compartmental concentrations are obtained by only constraining the sum of metabolites that occur in multiple compartments, thereby leaving the compartmental distribution of such metabolites free.

To find out how the applied thermodynamic constraints affect redox metabolism we analyzed all reactions involved in NADH redox metabolism in more detail. Yeast has multiple ways to oxidize NADH and to pass the electrons to the electron transport chain, both in the cytosol and the mitochondrion [30]. In the cytosol, the mitochondrial membrane bound external NADH dehydrogenase 1 and 2 complex (Nde1p/Nde2p), and the system involving glycerol-3-phosphate dehydrogenases (Gut2p, Gpd1p/Gpd2p), are mainly responsible for oxidation of NADH [31]. In the mitochondrion, NADH is oxidized by the internal NADH dehydrogenase (Ndi1p) [32]. Although yeast has a transporter that allows transport of *de novo* synthesized 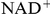 into the mitochondrion, transport of NADH between the cytosol and the mitochondrion does not occur [33]. Therefore, NADH is either oxidized in the compartment where it was produced, or it is transported through a shuttle mechanism, such as the ethanol-acetaldehyde redox shuttle [27], [34]. It is generally unclear what the relative contributions of these systems are to the overall oxidation of NADH, particularly of the NADH produced in the cytosol, despite thorough investigation [31]. Also, it is difficult to determine their relative contribution, because upon deletion of either one system, the other system can (partially) support the oxidation requirements.

**Figure 6:**
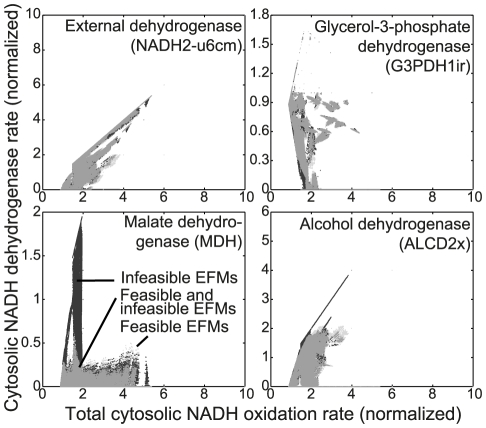
Cytosolic NADH oxidation rates of specific reactions versus the total cytosolic NADH oxidation rate for EFMs. The reaction name is shown on each plot, with the reaction name in the metabolic model in brackets. Every dot represents one or more EFMs at the respective combination of oxidation rates. A grey shade represents a combination where both feasible and infeasible EFMs are present. The rates are normalized to the glucose uptake rate of each EFM.

Thus, we determined the rates of the various cytosolic NADH oxidation mechanisms in every EFM and asked whether there is a difference between feasible and infeasible EFMs. Fig. 6 shows the total cytosolic NADH oxidation rate in each EFM against the respective NADH oxidation rate of a particular reaction, in the feasible and infeasible EFMs. It can be seen that the highest capacity to oxidize NADH comes from the external dehydrogenase complex (NADH2-u6m in Fig. 6), because only the external dehydrogenases could facilitate the complete oxidation, and indeed it was earlier found that the external dehydrogenase complex is mainly responsible for NADH oxidation in the cytosol [31]. The maximum total cytosolic NADH oxidation rate of 5.5 mol NADH/mol glucose cannot be reached by any of the other reactions. Particularly, malate dehydrogenase (MDH) and alcohol dehydrogenase (ALCD2x) are limited in their oxidation rate, with a maximum rate of 0.5 mol NADH/mol glucose for MDH, and a maximum rate of 4 mol/mol for ALCD2x, where only few EFMs have a rate higher than 2.2 mol/mol. The limits we find show that, on a quantitative level, redox metabolism is indeed affected by thermodynamic constraints. Specifically, we can see that from the known processes involved in the oxidation of NADH, the system providing the largest capacity for NADH oxidation is through the external dehydrogenase complex.

## Discussion

In this work, we demonstrated that an in vivo thermodynamically feasible metabolic flux distribution is only composed of thermodynamically feasible elementary flux modes. This EFM property allowed us to integrate EFMs and NET analysis into a novel approach to study the system-level properties of complex metabolic networks on the basis of quantitative metabolome data. As exemplified with a compartmentalized model of central metabolism in *S. cerevisiae* and cell-averaged metabolome data generated under glucose batch conditions, 46% of the 71.3 million EFMs were found thermodynamically infeasible, leading to direct insights into reaction directionalities, to constraints in several metabolic rates, and to the identification of reaction patterns that must be inactive due to a thermodynamic infeasibility.

This work builds on earlier work that integrated FBA, thermodynamics and quantitative metabolite data [14], [15], and extends it by using EFMs, allowing us to identify the reasons underlying the infeasibilities without making a priori assumptions on the metabolic objectives of the cell, such as maximization of biomass production, as is the case with FBA. Additionally, in our study we considered a compartmented metabolic network of *S. cerevisiae* to analyze cell-averaged metabolite data. Notably, the results we obtain are directly related to compartmentation, as can be seen from the identified infeasibility patterns that involve both compartments. The identified infeasibility of the ethanol-acetaldehyde redox shuttle has been previously identified using NET analysis [12] and manual consideration of the system. In this work we demonstrate that by using the flux patterns that are obtained from the EFMs we systematically identify such infeasibility patterns. Although the system-level constraints and their underlying causes can be rationalized without using EFMs, we need the generated EFMs to determine the infeasible patterns that are part of a stoichiometrically balanced flux distribution. In addition, because the number of EFMs can be considered approximately proportional to the flux solution space, we find that roughly half of the flux solution space is thermodynamically infeasible due to systems of reaction activities.

With the recently developed new group contribution method to estimate thermodynamic properties on a genome-scale [35], the recently increased availability in thermodynamic properties through experimental methods [36], the advances in quantitative metabolomics [37] and the now available methods to calculate EFMs also for large stoichiometric network [38], [39], we envision that the here presented approach will be helpful to shed light on metabolic flux physiology in more complex metabolic system such as higher cells simultaneously growing on multiple carbon substrates, where the applicability of classical flux analysis methods are still rather limited.

## Materials and Methods

### Experimental data

We used experimental data on metabolite concentrations for *Saccharomyces cerevisiae* obtained from four independent experiments with at least two replicates [40]–[42] with equal growth medium but under different cultivation conditions (bioreactor, shake flask, 96-well). Based on the data from the independent experiments, we constructed a consensus data set, where for each metabolite a minimum and maximum concentration was defined. The minimum and maximum concentrations were determined from all the replicates of measurements for each metabolite. To reduce the effect of outliers on the ranges, when more than 3 replicates were available, we removed the values higher than the third quartile +1.5 IQR (inter quartile range), and values lower than the first quartile −1.5 IQR. Physiological data was obtained for *S. cerevisiae* on glucose as carbon source from one of the four experiments. In Dataset S2 we describe the details of the experimental conditions of the data sets, the obtained concentration ranges and physiological data.

### Stoichiometric network

The stoichiometric metabolic network model describes the core central carbon metabolism of *S. cerevisiae* in the cytosol and mitochondrion with 230 reactions and 218 metabolites (see Dataset S3), and was developed on the basis of the genome-scale metabolic model iND750 that contains 1149 reactions and 646 metabolites [21]. For our model, we selected the cytosolic and mitochondrial reactions belonging to glycolysis/gluconeogenesis, pentose-phosphate pathway (PPP), TCA cycle, anaplerosis, pyruvate metabolism, and oxidative phosphorylation. The reversibility of each reaction was taken from Duarte et al. [21]. A cytosolic malate synthase was added to complement the glyoxylate shunt in the cytosol [43]. A citrate synthase was added to the cytosol since this is supported by localization studies [44].

To allow the model to synthesize all amino acids that are required for biomass, we added the following pathways: For L-alanine, two biosynthetic routes from pyruvate were included: cytosolic and mitochondrial alanine transaminase reactions, which were assumed to solely produce but not degrade L-alanine [45], [46]. Furthermore, L-glutamate could be produced via three alternative pathways: cytosolic or mitochondrial NADP-dependent glutamate dehydrogenase from alpha-ketoglutarate or mitochondrial NAD-dependent glutamate synthase from alpha-ketoglutarate and glutamine [47]. For glycine synthesis, we implemented three pathways such that it could be synthesized in the mitochondria via (i) alanine-glyoxylate transaminase [48], or in the cytosol by (ii) glycine hydroxymethyltransferase from serine [49], or (iii) from L-threonine via threonine aldolase [50]. As the latter reaction was assumed to be reversible, it could also be used to produce L-threonine, and such it constitutes a second possibility to produce L-threonine next to the linear pathway from L-aspartate. For all other amino acids, the model contains only one linear cytosolic pathway consisting of consecutive enzymatic reaction steps. Here, no alternative paths exist or they are excluded based on biochemical literature as it was done also to construct models for 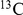-based flux analysis [45], [46].

The model further includes transport reactions across the mitochondrial membrane for metabolites that participate in reactions in both the cytosol and the mitochondria. Additional transport reactions that were not contained in iND750 (i.e. for L-glutamate, alpha-ketoglutarate, homocitrate, glyoxylate, and 2-oxobutanoate) were added to properly connect additionally included alternative pathways for amino acid synthesis to the metabolic network.

The biomass composition was adopted from iND750 besides that trehalose and glycogen were discarded since carbohydrate storage was not considered in our model. Lumped reactions for synthesis of the remaining biomass constituents, i.e. lipids, nucleotides, and cell wall components from the corresponding precursors were determined based on the biomass composition as provided in iND750. In the model, carbon molecules that can be exchanged with the environment are glucose, glycerol, pyruvate, acetate, ethanol, succinate, and 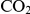.

The model is not proton balanced. The reason for this is that it is close to impossible to do the proton balancing correctly (e.g., for transport reactions). Thus, we did not want to add any potentially wrong constraints on the model and therefore did not account for proton balancing, with one exception. We only balanced the protons around the respiratory chain by replacing the cytosolic protons produced and consumed in the reactions CYOR_u6m, CYOOm and ATPS3m by a unique species “hcyt”. As a result, ATP generated in ATPS3m can only occur through the respiratory chain.

### Thermodynamic calculations

With NET analysis we can determine the feasibility of a flux distribution based on ranges for the concentrations of the involved metabolites. A flux distribution is thermodynamically infeasible when one or more reaction activities conflict with the calculated Gibbs energy of reaction range(s). Conversely, a flux distribution is feasible when no conflicts are found. It is important to note that a metabolite concentration is constrained in NET analysis by any reaction that has the metabolite as a reactant. Therefore, a flux distribution can be infeasible due to propagated constraints in a pathway. The NET analysis implementation constrains metabolite concentrations of metabolites that occur in multiple compartments as a sum of the compartment specific concentrations, corrected for their volume. Therefore, the compartmental distribution of such metabolites is left free. The compartmental volume fractions of the cytosol and mitochondrion are set to 0.35 and 0.1, respectively. The NET analysis approach is similar to other thermodynamic analysis approaches [14], [15]. A main difference from other approaches is that with NET analysis we aim at checking flux distributions for thermodynamic feasibility, and at estimating ranges of Gibbs reaction energies and metabolite concentrations.

For NET analysis we used the concentration ranges defined from the experimental data. For all other metabolites in the network we assumed a default range with a minimum concentration of 0.0001 mM and a maximum of 120 mM, except for carbon dioxide (“co2tot”), phosphate (“pi”) and diphosphate (“ppi”) that were constrained to a range of 1 mM to 100 mM [51], [52], and oxygen (“o2”) that was constrained to 0.001 mM to 0.1 mM [53]. By using such large metabolite concentration ranges we account for the noise in the metabolite concentration data and uncertainties in Gibbs energies of formation. Typical uncertainties in formation energies are in the order of 0.02–2 kJ/mol [35], which are overshadowed by variations in metabolite concentration data. The compartmental pH values were set to 5, 6.5 and 7 for the external, cytosolic and mitochondrial environment, respectively [54], [55]. The ionic strength was set to 0.15 M for all compartments. For the correct consideration of transport thermodynamics in NET analysis, we defined the specific transported species for each transport reaction where possible, and calculated transport reaction 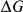 values according to Jol et al. [56].

### EFM generation, feasibility analysis and resolving infeasibility patterns

Computations for FVA, NET analysis and EFM generation were done using MATLAB (The Mathworks). For optimization of FVA problems we used the LINDO API library (LINDO Systems Inc.) and for NET analysis we used anNET [23] in combination with the LINDO global solver. To generate EFMs we used the Java implementation from Terzer and Stelling [19] on a quad-core system (3 GHz) with 128 GB memory. To test the thermodynamic feasibility of each EFM we used anNET, which was modified to run in an automated way on a cluster of computers encompassing on average 60 CPUs (3 GHz). Testing EFMs for thermodynamic feasibility was computationally intensive and took approximately 14 days.

To find the reaction activity patterns that cause infeasibility, we considered each infeasible EFM separately and performed an iterative analysis. In NET analysis of the EFM, we removed consecutively each reaction's activity constraint from the NET analysis optimization and determined the feasibility. If the activity pattern became thermodynamically feasible, the reaction activity that was removed was identified as part of the pattern. Then we continued with removing the next reaction activity, while keeping the activities that were identified as part of the pattern. We continued this process for all the reaction activities. This process led to a set of reaction activities, which is a subset of the activities in the analyzed EFM, of which the removal of one activity leads to a feasible system. The set of reaction activities is only infeasible as a whole system, and no single reaction can be marked infeasible by itself. All possible infeasible reaction patterns may not be found when multiple patterns are present in an EFM, because the order of reaction activity removal determines which pattern is found. The obtained infeasible patterns cover all the infeasible EFMs.

### Flux balance analysis

Flux balance analysis with maximization of biomass production, maximization of ATP production and maximization of the ratio of ATP production over the sum of squared fluxes was performed according to Schütz et al. [28] with the physiological data used for FVA as constraints. The computations were done using MATLAB (The Mathworks) using the LINDO API library (LINDO Systems Inc.) for optimization.

## Supporting Information

Dataset S1 Overview showing for each model reaction at which stage during the analysis a directionality constraint was added.
(PDF)

Dataset S2 Details regarding the experimental data used in this study.
(PDF)

Dataset S3 The used metabolic network model in SBML format.
(XML)

Text S1 Mathematic proof showing that a flux distribution containing an infeasible EFM is always infeasible.
(PDF)

Text S2 Additional results regarding flux balance analysis showing the obtained patterns correspond with known operating principles.
(PDF)
