# Supplementary material for: System-Level Insights into Yeast Metabolism by Thermodynamic Analysis of Elementary Flux Modes
Source: PLoS Comput Biol. 2012 Mar 1;8(3):e1002415. doi: 10.1371/journal.pcbi.1002415 (PMC3296127; doi:10.1371/journal.pcbi.1002415)
Supplement: Dataset S1 — Overview showing for each model reaction at which stage during the analysis a directionality constraint was added. (PDF) [file pcbi.1002415.s001.pdf]

Dataset S1. Model and constraint addition

In this supplement we show for each model reaction at which stage during the analysis a directionality constraint was added. For reactions, the compartment is indicated between brackets, where [e] indicates external, [c] indicates cytosol and [m] indicates mitochondrion. Compartment indicators before a reaction means that the complete reaction occurs in the respective compartment. For five stages where the reaction directions are constrained the reaction direction is shown, where 0 means reversible, -1 means backward and 1 means forward. "Initial" are reaction directions as set in the genome-scale model, "A-priori" are reaction directions that were set beforehand, "After FVA" are reaction directions obtained from FVA, "After NETa" are reaction directions obtained from NET analysis with the reaction activities from FVA, and "Feasible EFMs" are reaction directions that are possible in the feasible set of EFMs, where 0 means both forward and backward reaction directions occur in the EFMs.

			Direction				
	Name	Reaction	Initial	A-priori	After FVA	After NETa	Feasible EFMs
1	ASPTA	[c] akg + asp-L <==> glu-L + oaa	0	0	-1	-1	-1
2	ASPKi	[c] asp-L + atp --> 4pasp + adp	1	1	1	1	1
3	ASNS1	[c] asp-L + atp + gln-L + h2o --> amp + asn-L + glu-L + h + ppi	1	1	1	1	1
4	ASADi	[c] 4pasp + h + nadph --> aspsa + nadp + pi	1	1	1	1	1
5	ALATA_Lm	[m] glu-L + pyr --> akg + ala-L	1	1	1	1	1
6	ALATA_L	[c] glu-L + pyr --> akg + ala-L	1	1	1	1	1
7	PPCK	[c] atp + oaa + h2o --> adp + co2tot + pep	1	1	1	1	1
8	PC	[c] atp + co2tot + pyr --> adp + h + oaa + pi	1	1	1	1	1
9	ME2m	[m] mal-L + nadp + h2o --> co2tot + nadph + pyr	1	1	1	1	1
10	ICL	[c] icit --> glx + succ	1	1	1	1	1
11	FBP	[c] fdp + h2o --> f6p + pi	1	1	1	1	1
12	P5CR	[c] 1pyr5c + (2) h + nadph --> nadp + pro-L	1	1	1	1	1
13	ORNTACim	[m] acorn + glu-L --> acglu + orn	1	1	1	1	1
14	OCBTi	[c] cbp + orn --> citr-L + h + pi	1	1	1	1	1
15	GLU5K	[c] atp + glu-L --> adp + glu5p	1	1	1	1	1
16	G5SD	[c] glu5p + h + nadph --> glu5sa + nadp + pi	1	1	1	1	1
17	G5SADr	[c] glu5sa <==> 1pyr5c + h + h2o	0	0	1	1	1
18	CBPS	[c] (2) atp + gln-L + h2o + co2tot --> (2) adp + cbp + glu-L + (2) h + pi	1	1	1	1	1
19	ARGSSr	[c] asp-L + atp + citr-L <==> amp + argsuc + h + ppi	0	0	1	1	1
20	ARGSL	[c] argsuc <==> arg-L + fum	0	0	1	1	1
21	AGPRim	[m] acg5p + h + nadph --> acg5sa + nadp + pi	1	1	1	1	1
22	ACOTAim	[m] acg5sa + glu-L --> acorn + akg	1	1	1	1	1
23	ACGKm	[m] acglu + atp --> acg5p + adp	1	1	1	1	1
24	SUCOAS1m	[m] atp + coa + succ <==> adp + pi + succoa	0	0	0	0	0
25	SUCD2_u6	[m] q6 + succ <==> fum + q6h2	0	0	0	0	0
26	ICDHy	[c] icit + nadp + h2o --> akg + co2tot + nadph	1	1	1	1	1
27	ICDHxm	[m] icit + nad + h2o --> akg + co2tot + nadh	1	1	1	1	1
28	CSm	[m] accoa + h2o + oaa --> cit + coa + h	1	1	1	1	1
29	AKGDam	[m] akg + coa + h2o + nad <==> co2tot + nadh + succoa	0	0	0	0	0
30	ACONTm	[m] cit <==> icit	0	0	0	0	0
31	ACONT	[c] cit <==> icit	0	0	0	0	0
32	SULR	[c] (3) h2o + h2s + (3) nadp <==> (5) h + (3) nadph + so3	0	0	-1	-1	-1
33	SLFAT	[c] adp + h + so4 <==> aps + pi	0	0	1	1	1
34	PAPSR	[c] paps + nadph + h --> (2) h + pap + so3 + nadp	1	1	1	1	1
35	BPNT	[c] h2o + pap --> amp + pi	1	1	1	1	1
36	ADSK	[c] aps + atp --> adp + h + paps	1	1	1	1	1
37	MTHFR3	[c] h + mlthf + nadph --> 5mthf + nadp	1	1	1	1	1
38	MTHFD	[c] mlthf + nadp <==> h + methf + nadph	0	0	1	1	1
39	MTHFC	[c] h2o + methf <==> 10fthf	0	0	1	1	1
40	FTHFL	[c] atp + for + thf <==> 10fthf + adp + pi	0	0	0	0	0
41	GLUDy	[c] glu-L + h2o + nadp <==> akg + h + nadph + nh4	0	0	-1	-1	-1
42	GLNS	[c] atp + glu-L + nh4 --> adp + gln-L + h + pi	1	1	1	1	1
43	ANS	[c] chor + gln-L --> anth + glu-L + h + pyr	1	1	1	1	1
44	GLYK	[c] atp + glyc --> adp + glyc3p + h	1	1	1	1	1
45	G3PT	[c] glyc3p + h2o --> glyc + pi	1	1	1	1	1
46	G3PD1ir	[c] dhap + h + nadh --> glyc3p + nad	1	1	1	1	1
47	ALCD2m	[m] etoh + nad <==> acald + h + nadh	0	0	0	0	0
48	PSP_L	[c] h2o + pser-L --> pi + ser-L	1	1	1	1	1
49	PSERT	[c] 3php + glu-L --> akg + pser-L	1	1	1	1	1
50	PGCD	[c] 3pg + nad --> 3php + h + nadh	1	1	1	1	1
51	HSK	[c] atp + hom-L --> adp + h + phom	1	1	1	1	1
52	HSDyi	[c] aspsa + h + nadph --> hom-L + nadp	1	1	1	1	1
53	GHMT2r	[c] ser-L + thf <==> gly + h2o + mlthf	0	0	1	1	1
54	CYSTS	[c] hcys-L + ser-L --> cyst-L + h2o	1	1	1	1	1
55	TPI	[c] dhap <==> g3p	0	0	0	0	0
56	PYK	[c] adp + h + pep --> atp + pyr	1	1	1	1	1
57	PGM	[c] 2pg <==> 3pg	0	0	-1	-1	-1
58	PGK	[c] 3pg + atp <==> 13dpg + adp	0	0	-1	-1	-1
59	PGI	[c] g6p <==> f6p	0	0	1	1	1
60	PFK	[c] atp + f6p --> adp + fdp + h	1	1	1	1	1
61	PDHm	[m] coa + nad + pyr + h2o --> accoa + co2tot + nadh	1	1	1	1	1

62	HEX1	[c] atp + glc-D --> adp + g6p + h	1	1	1	1	1
63	GAPD	[c] g3p + nad + pi <==> 13dpg + h + nadh	0	0	1	1	1
64	FBA	[c] fdp <==> dhap + g3p	0	0	1	1	1
65	ENO	[c] 2pg <==> h2o + pep	0	0	1	1	1
66	PRPPS	[c] atp + r5p <==> amp + h + prpp	0	0	1	1	1
67	PRMICIi	[c] prfp --> prlp	1	1	1	1	1
68	PRATPP	[c] h2o + prbatp --> h + ppi + prbamp	1	1	1	1	1
69	PRAMPC	[c] h2o + prbamp --> prfp	1	1	1	1	1
70	IGPDH	[c] eig3p --> h2o + imacp	1	1	1	1	1
71	IG3PS	[c] gln-L + prlp --> aicar + eig3p + glu-L + h	1	1	1	1	1
72	HSTPT	[c] glu-L + imacp --> akg + hisp	1	1	1	1	1
73	HISTP	[c] h2o + hisp --> histd + pi	1	1	1	1	1
74	HISTD	[c] h2o + histd + (2) nad --> (3) h + his-L + (2) nadh	1	1	1	1	1
75	ATPPRT	[c] atp + prpp --> ppi + prbatp	1	1	1	1	1
76	FDH	[c] for + nad + h2o --> co2tot + nadh	1	1	1	1	1
77	METS	[c] 5mthf + hcys-L --> met-L + thf	1	1	1	1	1
78	HSERTA	[c] accoa + hom-L <==> achms + coa	0	0	1	1	1
79	CYSTGL	[c] cyst-L + h2o --> 2obut + cys-L + nh4	1	1	1	1	1
80	AHSERL2	[c] achms + h2s --> ac + h + hcys-L	1	1	1	1	1
81	ADK1m	[m] amp + atp <==> (2) adp	0	0	1	1	1
82	ADK1	[c] amp + atp <==> (2) adp	0	0	1	1	1
83	PPAm	[m] h2o + ppi --> h + (2) pi	1	1	1	1	1
84	PPA	[c] h2o + ppi --> h + (2) pi	1	1	1	1	1
85	NADH2-u6	[m] h + nadh + q6 --> nad + q6h2	1	1	1	1	1
86	NADH2-u6	h[c] + nadh[c] + q6[m] --> nad[c] + q6h2[m]	1	1	1	1	1
87	MDHm	[m] mal-L + nad <==> h + nadh + oaa	0	0	1	1	1
88	MDH	[c] mal-L + nad <==> h + nadh + oaa	0	0	0	0	0
89	FUMm	[m] fum + h2o <==> mal-L	0	0	0	0	0
90	FUM	[c] fum + h2o <==> mal-L	0	0	1	1	1
91	CYOR_u6m	(2) ficytc[m] + (1.5) h[m] + q6h2[m] --> (2) focytc[m] + (1.5) h[c] + q6[m]	1	1	1	1	1
92	CYOOm	(4) focytc[m] + (6) h[m] + o2[m] --> (4) ficytc[m] + (6) h[c] + (2) h2o[m]	1	1	1	1	1
93	ATPS3m	adp[m] + (3) h[c] + pi[m] --> atp[m] + (3) h[m] + h2o[m]	1	1	1	1	1
94	TKT2	[c] e4p + xu5p-D <==> f6p + g3p	0	1	1	1	1
95	TKT1	[c] r5p + xu5p-D <==> g3p + s7p	0	1	1	1	1
96	TALA	[c] g3p + s7p <==> e4p + f6p	0	1	1	1	1
97	RPI	[c] r5p <==> ru5p-D	0	-1	-1	-1	-1
98	RPE	[c] ru5p-D <==> xu5p-D	0	1	1	1	1
99	PGL	[c] 6pgl + h2o --> 6pgc + h	1	1	1	1	1
100	GND	[c] 6pgc + nadp + h2o --> co2tot + nadph + ru5p-D	1	1	1	1	1
101	G6PDH2	[c] g6p + nadp --> 6pgl + h + nadph	1	1	1	1	1
102	PYRDC	[c] h + pyr + h2o --> acald + co2tot	1	1	1	1	1
103	ALCD2x	[c] etoh + nad <==> acald + h + nadh	0	0	0	0	0
104	ACSm	[m] ac + atp + coa --> accoa + amp + ppi	1	1	1	1	1
105	ACS	[c] ac + atp + coa --> accoa + amp + ppi	1	1	1	1	1
106	ACOAH	[c] ac + coa + h <==> accoa + h2o	0	0	0	-1	-1
107	THRS	[c] h2o + phom --> pi + thr-L	1	1	1	1	1
108	THRD_Lm	[m] thr-L --> 2obut + nh4	1	1	1	1	1
109	THRA	[c] acald + gly <==> thr-L	0	0	0	0	0
110	SACCD2	[c] h2o + nad + saccrp-L <==> akg + h + lys-L + nadh	0	0	1	1	1
111	SACCD1	[c] L2aadp6sa + glu-L + h + nadph <==> h2o + nadp + saccrp-L	0	0	1	1	1
112	OXAGm	[m] h + oxag + h2o <==> 2oxoadp + co2tot	0	0	1	1	1
113	MCITDm	[m] hcit <==> b124tc + h2o	0	0	1	1	1
114	HICITDm	[m] hicit + nad <==> h + nadh + oxag	0	0	1	1	1
115	HACNHm	[m] b124tc + h2o <==> hicit	0	0	1	1	1
116	AATA	[c] 2oxoadp + glu-L <==> L2aadp + akg	0	0	1	1	1
117	AASAD1	[c] L2aadp + atp + h + nadph --> L2aadp6sa + amp + nadp + ppi	1	1	1	1	1
118	TYRTA	[c] akg + tyr-L <==> 34hpp + glu-L	0	0	-1	-1	-1
119	TRPS1	[c] 3ig3p + ser-L --> g3p + h2o + trp-L	1	1	1	1	1
120	SHKK	[c] atp + skm --> adp + h + skm5p	1	1	1	1	1
121	SHK3D	[c] 3dhsk + h + nadph --> nadp + skm	1	1	1	1	1
122	PSCVTi	[c] pep + skm5p --> 3psme + pi	1	1	1	1	1
123	PRAIi	[c] pran --> 2cpr5p	1	1	1	1	1
124	PPNDH	[c] h + pphn --> co2tot + phpyr	1	1	1	1	1
125	PPND2	[c] nadp + pphn + h2o --> 34hpp + co2tot + nadph	1	1	1	1	1
126	PHETA1	[c] akg + phe-L <==> glu-L + phpyr	0	0	-1	-1	-1
127	IGPS	[c] 2cpr5p + h --> 3ig3p + co2tot	1	1	1	1	1
128	DHQTi	[c] 3dhq --> 3dhsk + h2o	1	1	1	1	1
129	DHQS	[c] 2dda7p --> 3dhq + pi	1	1	1	1	1
130	DDPA	[c] e4p + h2o + pep --> 2dda7p + pi	1	1	1	1	1
131	CHORS	[c] 3psme --> chor + pi	1	1	1	1	1
132	CHORM	[c] chor --> pphn	1	1	1	1	1
133	ANPRT	[c] anth + prpp --> ppi + pran	1	1	1	1	1
134	ALDD2ym	[m] acald + h2o + nadp --> ac + (2) h + nadph	1	1	1	1	1
135	ALDD2y	[c] acald + h2o + nadp --> ac + (2) h + nadph	1	1	1	1	1
136	ALDD2xm	[m] acald + h2o + nad --> ac + (2) h + nadh	1	1	1	1	1
137	VALTA	[c] akg + val-L <==> 3mob + glu-L	0	0	-1	-1	-1
138	OMCDC	[c] 3c4mop + h + h2o --> 4mop + co2tot	1	1	1	1	1
139	LEUTA	[c] akg + leu-L <==> 4mop + glu-L	0	0	-1	-1	-1
140	KARA2im	[m] 2ahbut + h + nadph --> 23dhmp + nadp	1	1	1	1	1
141	KARA1im	[m] alac-S + h + nadph --> 23dhmb + nadp	1	1	1	1	1
142	IPPS	[c] 3mob + accoa + h2o --> 3c3hmp + coa + h	1	1	1	1	1
143	IPPMIb	[c] 2ippm + h2o <==> 3c3hmp	0	0	-1	-1	-1
144	IPPMIa	[c] 3c2hmp <==> 2ippm + h2o	0	0	-1	-1	-1
145	IPMD	[c] 3c2hmp + nad --> 3c4mop + h + nadh	1	1	1	1	1

146	ILETA	[c] akg + ile-L <==> 3mop + glu-L	0	0	-1	-1	-1
147	DHAD2m	[m] 23dhmp --> 3mop + h2o	1	1	1	1	1
148	DHAD1m	[m] 23dhmb --> 3mob + h2o	1	1	1	1	1
149	ACLSm	[m] h + (2) pyr + h2o --> alac-S + co2tot	1	1	1	1	1
150	ACHBSm	[m] 2obut + h + pyr + h2o --> 2ahbut + co2tot	1	1	1	1	1
151	GLUSxm	[m] akg + gln-L + h + nadh --> (2) glu-L + nad	1	1	1	1	1
152	AGTm	[m] ala-L + glx <==> gly + pyr	0	0	0	0	0
153	HCITS	[c] accoa + akg + h2o <==> coa + h + hcit	0	0	1	1	1
154	MALS	[c] accoa + glx + h2o <==> coa + h + mal-L	0	0	0	1	1
155	GLUDym	[m] glu-L + h2o + nadp <==> akg + h + nadph + nh4	0	0	0	0	0
156	CSc	[c] accoa + h2o + oaa --> cit + coa + h	1	1	1	1	1
157	SUCCt2r	h[e] + succ[e] <==> h[c] + succ[c]	0	0	-1	-1	-1
158	SO4ti	so4{-2}[e] --> so4{-2}[c]	1	1	1	1	1
159	PYRt2	pyr[e] <==> pyr[c]	0	0	-1	-1	-1
160	PIt2r	h[e] + pi{-1}[e] <==> h[c] + pi{-1}[c]	0	0	1	1	1
161	O2t	o2[e] <==> o2[c]	0	0	1	1	1
162	NH4t	nh4{+1}[e] <==> nh4{+1}[c]	0	0	1	1	1
163	H2Ot	h2o[e] <==> h2o[c]	0	0	0	0	0
164	GLYCt	glyc[c] <==> glyc[e]	0	0	1	1	1
165	GLCt1	glc-D[e] --> glc-D[c]	1	1	1	1	1
166	ETOHt	etoh[e] <==> etoh[c]	0	0	-1	-1	-1
167	CO2t	co2tot[e] <==> co2tot[c]	0	0	-1	-1	-1
168	ATPS	atp[c] + h2o[c] --> adp[c] + h[e] + pi[c]	1	1	1	1	1
169	ACt2r	ac{0}[e] <==> ac{0}[c]	0	0	-1	-1	-1
170	THRt2m	thr-L{0}[c] <==> thr-L{0}[m]	0	0	1	1	1
171	SUCFUMtm	fum{-2}[m] + succ{-2}[c] --> fum{-2}[c] + succ{-2}[m]	1	1	1	1	1
172	SUCCtm	pi{-2}[m] + succ{-2}[c] --> pi{-2}[c] + succ{-2}[m]	1	1	1	1	1
173	PYRt2m	h[c] + pyr{-1}[c] <==> h[m] + pyr{-1}[m]	0	0	1	1	1
174	PIt2m	h[c] + pi{-1}[c] <==> h[m] + pi{-1}[m]	0	0	0	0	0
175	ORNt3m	orn[m] <==> orn[c]	0	0	1	1	1
176	OAAt2m	(2) h[c] + oaa{-2}[c] <==> (2) h[m] + oaa{-2}[m]	0	0	0	-1	Inactive
177	O2tm	o2[c] <==> o2[m]	0	0	1	1	1
178	NH4tm	nh4{+1}[c] <==> nh4{+1}[m]	0	0	0	0	0
179	MALtm	mal-L{-2}[c] + pi{-2}[m] <==> mal-L{-2}[m] + pi{-2}[c]	0	0	0	0	0
180	H2Otm	h2o[c] <==> h2o[m]	0	0	0	0	0
181	GLYt2m	gly{0}[c] <==> gly{0}[m]	0	0	0	0	0
182	GLUt2m	glu-L{-1}[c] + h[c] <==> glu-L{-1}[m] + h[m]	0	0	0	0	0
183	ETOHtm	etoh[c] <==> etoh[m]	0	0	0	0	0
184	CO2tm	co2tot[c] <==> co2tot[m]	0	0	-1	-1	-1
185	CITtcm	cit{-3}[c] + icit{-3}[m] <==> cit{-3}[m] + icit{-3}[c]	0	0	0	0	0
186	CITtam	cit{-3}[c] + mal-L{-2}[m] <==> cit{-3}[m] + mal-L{-2}[c]	0	0	0	0	0
187	ATPtm-H	adp{-3}[c] + atp{-4}[m] --> adp{-3}[m] + atp{-4}[c]	1	1	1	1	1
188	ACtm	ac{0}[c] <==> ac{0}[m]	0	0	0	0	0
189	3MOPtm	3mop[c] <==> 3mop[m]	0	0	-1	-1	-1
190	3MOBtm	3mob[c] <==> 3mob[m]	0	0	-1	-1	-1
191	2OXOADPt	2oxoadp[m] + akg[c] --> 2oxoadp[c] + akg[m]	1	1	1	1	1
192	GLNt2m	gln-L{0}[c] <==> gln-L{0}[m]	0	0	1	1	1
193	2OBUTtm	2obut[c] <==> 2obut[m]	0	0	1	1	1
194	ALAt2m	ala-L{0}[c] <==> ala-L{0}[m]	0	0	0	0	0
195	HCITtm	hcit[c] <==> hcit[m]	0	0	1	1	1
196	GLXtm	glx{0}[c]<==> glx{0}[m]	0	0	0	0	0
197	ACALDtm	acald[c] <==> acald[m]	0	0	0	0	1
198	ac[e]_EX	ac[e] <==>	0	0	1	1	1
199	co2[e]_EX	co2tot[e] <==>	0	0	1	1	1
200	etoh[e]_EX	etoh[e] <==>	0	0	1	1	1
201	glc-D[e]_E	glc-D[e] <==>	0	0	-1	-1	-1
202	glyc[e]_EX	glyc[e] <==>	0	0	1	1	1
203	h[e]_EX	h[e] <==>	0	0	0	0	0
204	h2o[e]_EX	h2o[e] <==>	0	0	0	0	0
205	nh4[e]_EX	nh4[e] <==>	0	0	-1	-1	-1
206	o2[e]_EX	o2[e] <==>	0	0	-1	-1	-1
207	pi[e]_EX	pi[e] <==>	0	0	-1	-1	-1
208	pyr[e]_EX	pyr[e] <==>	0	0	1	1	1
209	so4[e]_EX	so4[e] <==>	0	0	-1	-1	-1
210	succ[e]_EX	succ[e] <==>	0	0	1	1	1
211	aicarSYN	10fthf[c] + asp-L[c] + (5) atp[c] + co2tot[c] + (2) gln-L[c] + gly[c] + h2o[c] + r5p[c] --> (4) adp[c] + aicar[c] + amp[c] + fum[c] + (2) glu-L[c] + (8) h[c] + (4) pi[c] + ppi[c] + thf[c]	1	1	1	1	1
212	ampSYN	10fthf[c] + aicar[c] + asp-L[c] + atp[c] --> adp[c] + amp[c] + fum[c] + (2) h[c] + h2o[c] + pi[c] + thf[c]	1	1	1	1	1
213	dampSYN	amp[c] + h[c] + nadph[c] --> damp[c] + h2o[c] + nadp[c]	1	1	1	1	1
214	gmpSYN	10fthf[c] + aicar[c] + atp[c] + gln-L[c] + h2o[c] + nad[c] --> amp[c] + glu-L[c] + gmp[c] + (3) h[c] + nadh[c] + ppi[c] + thf[c]	1	1	1	1	1
215	dgmpSYN	gmp[c] + h[c] + nadph[c] --> dgmp[c] + h2o[c] + nadp[c]	1	1	1	1	1
216	umpSYN	asp-L[c] + (3) atp[c] + gln-L[c] + (0.5) o2[c] + r5p[c] --> (2) adp[c] + amp[c] + glu-L[c] + (2) h[c] + (2) pi[c] + ppi[c] + ump[c]	1	1	1	1	1
217	cmpSYN	atp[c] + nh4[c] + ump[c] --> adp[c] + cmp[c] + (2) h[c] + pi[c]	1	1	1	1	1
218	dcmpSYN	cmp[c] + h[c] + nadph[c] --> dcmp[c] + h2o[c] + nadp[c]	1	1	1	1	1
219	dtmpSYN	dcmp[c] + (2) h[c] + h2o[c] + mlthf[c] + nadph[c] --> dtmp[c] + nadp[c] + nh4[c] + thf[c]	1	1	1	1	1
220	13BDglcnS	atp[c] + g6p[c] --> 13BDglcn[c] + adp[c] + ppi[c]	1	1	1	1	1
221	mannanSYI	atp[c] + f6p[c] --> adp[c] + mannan[c] + ppi[c]	1	1	1	1	1

222	zymstSYN	(18) accoa[c] + (18) atp[c] + (11) h[c] + h2o[c] + (2) nad[c] + (26) nadph[c] + (10) o2[c] --> (18) adp[c] + (8) co2tot[c] + (18) coa[c] + for[c] + (2) nadh[c] + (26) nadp[c] + (6) pi[c] + (6) ppi[c] + zymst[c]	1	1	1	1	1
223	ergstSYN	(2) atp[c] + h[c] + met-L[c] + (3) nadph[c] + (2) o2[c] + zymst[c] --> adp[c] + amp[c] + ergst[c] + (2) h2o[c] + hcys-L[c] + (3) nadp[c] + pi[c] + ppi[c]	1	1	1	1	1
224	paSYN	(16.2) accoa[c] + (14.2) atp[c] + glyc3p[c] + (15.38) h[c] + (29.58) nadph[c] + (1.18) o2[c] --> (14.2) adp[c] + (16.2) coa[c] + (2.36) h2o[c] + (29.58) nadp[c] + pa_SC[c] + (14.2) pi[c]	1	1	1	1	1
225	psSYN	(2) atp[c] + pa_SC[c] + ser-L[c] --> (2) adp[c] + ppi[c] + ps_SC[c]	1	1	1	1	1
226	peSYN	h[c] + h2o[c] + ps_SC[c] --> co2tot[c] + pe_SC[c]	1	1	1	1	1
227	pcSYN	(6) atp[c] + (6) h2o[c] + (3) met-L[c] + pe_SC[c] --> (3) adp[c] + (3) amp[c] + (6) h[c] + (3) hcys-L[c] + pc_SC[c] + (3) pi[c] + (3) ppi[c]	1	1	1	1	1
228	ptd1inoSYN	(2) atp[c] + g6p[c] + h2o[c] + pa_SC[c] --> (2) adp[c] + pi[c] + ppi[c] + ptd1ino_SC[c]	1	1	1	1	1
229	triglycSYN	(8.1) accoa[c] + (7.1) atp[c] + (7.69) h[c] + (14.79) nadph[c] + (0.59) o2[c] + pa_SC[c] --> (7.1) adp[c] + (8.1) coa[c] + (0.18) h2o[c] + (14.79) nadp[c] + (8.1) pi[c] + triglyc_SC[c]	1	1	1	1	1
230	biomass	(1.1348) 13BDglcn + (0.4588) ala-L + (0.046) amp + (0.1607) arg-L + (0.1017) asn-L + (0.2975) asp-L + (59.276) atp + (0.0447) cmp + (0.0066) cys-L + (0.0036) damp + (0.0024) dcmp + (0.0024) dgmp + (0.0036) dtmp + (0.0007) ergst + (0.1054) gln-L + (0.3018) glu-L + (0.2904) gly + (0.046) gmp + (59.276) h2o + (0.0663) his-L + (0.1927) ile-L + (0.2964) leu-L + (0.2862) lys-L + (0.8079) mannan + (0.0507) met-L + (6e-006) pa_SC + (6e-005) pc_SC + (4.5e-005) pe_SC + (0.1339) phe-L + (0.1647) pro-L + (1.7e-005) ps_SC + (5.3e-005) ptd1ino_SC + (0.1854) ser-L + (0.02) so4 + (0.1914) thr-L + (6.6e-005) triglyc_SC + (0.0284) trp-L + (0.102) tyr-L + (0.0599) ump + (0.2646) val-L + (0.0015) zymst --> (59.276) adp + (58.7162) h + (59.305) pi	0	1	1	1	1
