# Supplementary material for: System-Level Insights into Yeast Metabolism by Thermodynamic Analysis of Elementary Flux Modes
Source: PLoS Comput Biol. 2012 Mar 1;8(3):e1002415. doi: 10.1371/journal.pcbi.1002415 (PMC3296127; doi:10.1371/journal.pcbi.1002415)
Supplement: Dataset S2 — Details regarding the experimental data used in this study. (PDF) [file pcbi.1002415.s002.pdf]

Dataset S2. Experimental data

We used experimental data on metabolite concentrations from four independent experiments (see Tab. 1). To construct a consensus data set where for each metabolite a range of the measured concentrations across all data sets is defined by a minimum and maximum concentration. The minimum and maximum concentrations where determined from all the replicates of the measurements for each metabolite after discarding the highest and lowest value as outliers.

Physiological data was obtained for *Saccharomyces cerevisiae* on glucose as carbon source, from the same experiment as data set D (Kümmel, 2008). The consumption rates was determined for glucose (12.4 mmol/gDW/h), and the production rates were determined for biomass (12.4 mmol/gDW/h), CO_2 (23.1 mmol/gDW/h), pyruvate (0.1 mmol/gDW/h), ethanol (16.6 mmol/gDW/h), acetate (0.6 mmol/gDW/h), glycerol (0.8 mmol/gDW/h) and succinate (<0.1 mmol/gDW/h).

Table 1: Data sets used to determine the metabolite concentration ranges for each metabolite

**Data set**	**Cultivation**	**Strain**	**Reference**
A	Shake-flask	FY4	Unpublished
B	Bioreactor	FY4	Kümmel (2008)
C	96-well plate	CEN.PK 113-7D	Ewald (2010)
D	Shake-flask	FY4	Fendt et al. (2010)

The metabolite concentration ranges as they are used in the NET analysis steps are shown in Tab. 2. The full names for the abbreviated metabolite names used here, and in Fig. 2 of the main text, are shown in Tab. 3. The abbreviated names correspond to the names used in the genome-scale metabolic model iND750 (Duarte et al., 2004).

Table 2: Metabolite concentration ranges as applied in NET analysis. For each metabolite the minimum (Min.), maximum (Max.) and the median value over all data points is given. Concentrations are in mM.

**Metabolite**	**Min.**	**Max.**	**Median**	**Metabolite**	**Min.**	**Max.**	**Median**
13dpg	0.139	0.168	0.155	hom-L	0.136	1.530	0.581
2pg+3pg	0.049	1.778	0.768	icit	0.075	0.251	0.171
3pg	0.336	0.415	0.369	ile-L	0.222	1.274	0.458
6pgc	0.099	1.421	0.670	leu-L	0.146	0.822	0.328
accoa	0.038	0.151	0.090	lys-L	3.144	4.075	3.187
adp	0.182	0.758	0.597	mal-L	0.176	2.003	0.810
akg	0.164	1.754	0.595	met-L	0.062	0.283	0.119
ala-L	5.015	12.850	8.315	nad	0.261	0.926	0.404
amp	0.066	0.226	0.151	nadh	0.092	0.594	0.402
arg-L	1.540	40.711	24.893	nadp	0.028	1.007	0.194
asn-L	0.417	5.370	2.490	nadph	0.021	0.109	0.055
asp-L	1.928	18.493	7.551	oaa	0.007	0.011	0.009
atp	0.502	3.885	2.670	orn	1.796	3.150	2.629
cit	0.247	1.743	0.718	pep	0.018	0.291	0.095
coa	0.012	0.036	0.027	phe-L	0.133	0.406	0.319
cys-L	0.147	0.252	0.199	pro-L	0.504	1.124	0.747
dhap	0.058	0.958	0.538	pyr	0.357	1.873	0.936
f6p	0.227	0.359	0.302	r5p	0.029	0.395	0.148
fdp	0.438	10.635	1.954	ru5p-D+xu5p-D	0.010	0.275	0.034
fum	0.076	0.318	0.200	ser-L	1.068	5.783	2.759
g3p	0.025	0.745	0.622	skm	0.003	0.038	0.015
g6p	0.779	4.314	1.767	succ	0.167	0.773	0.391
gln-L	4.891	89.660	33.473	succoa	1.043	2.547	1.795
glu-L	8.952	94.970	22.390	thr-L	1.130	13.647	8.282
glx	0.013	0.330	0.201	trp-L	0.023	0.069	0.037
gly	0.047	3.186	1.163	tyr-L	0.129	0.241	0.178
glyc3p	0.027	0.657	0.278	val-L	0.981	2.867	2.066
his-L	3.512	3.824	3.759				

Table 3: Metabolite abbreviations with their corresponding full names.

**Short name**	**Long name**	**Short name**	**Long name**
13dpg	1,3-phospho-glycerate	glyc	glycerol
2pg	2-phospho-glycerate	glyc3p	glycerol-3-phosphate
3pg	3-phospho-glycerate	his-L	L-histidine
6pgc	6-phospho-gluconate	hom-L	L-homoserine
6pgl	6-phospho-gluconolactone	icit	isocitrate
ac	acetate	ile-L	L-isoleucine
acald	acetaldehyde	leu-L	L-leucine
accoa	acetyl-CoA	lys-L	L-lysine
adp	ADP	mal-L	L-malate
akg	a-ketoglutarate	met-L	L-methionine
ala-L	L-alanine	nad	NAD
amp	AMP	nadh	NADH
arg-L	L-arginine	nadp	NADP
asn-L	L-asparagine	nadph	NADPH
asp-L	L-aspartate	oaa	oxaloacetate
atp	ATP	orn	ornithine
cit	citrate	pep	phosphoenolpyruvate
coa	coenzyme A	phe-L	L-phenylalanine
cys-L	L-cysteine	pro-L	L-proline
dhap	DHAP	pyr	pyruvate
e4p	erythrose-4-phosphate	r5p	ribose-5-phosphate
etoh	ethanol	ru5p-D	D-ribulose-5-phosphate
f6p	fructose-6-phosphate	s7p	sedoheptulose-7-phosphate
fdp	fructose-1,6-phosphate	ser-L	L-serine
fum	fumarate	skm	shikimate
g3p	glyceraldehyde-3-phosphate	succ	succinate
g6p	glucose-6-phosphate	succoa	succinyl-CoA
glc-D	glucose	thr-L	L-threonine
gln-L	L-glutamine	trp-L	L-tryptophan
glu-L	L-glutamate	tyr-L	L-tyrosine
glx	glyoxylate	val-L	L-valine
gly	glycine	xu5p-D	D-xylulose-5-phosphate

References

Duarte, N. C., Herrgård, M. J., and Palsson, B. O. (2004). Reconstruction and Validation of Saccharomyces cerevisiae iND750, a Fully Compartmentalized Genome-Scale Metabolic Model. *Genome Res.*, 14(7):1298–1309.

Ewald, J. C. (2010). *Unraveling Yeast's Response to its Environment by Novel Metabolomics Approaches.* PhD thesis, ETH Zürich.

Fendt, S.-M., Buescher, J. M., Rudroff, F., Picotti, P., Zamboni, N., and Sauer, U. (2010). Tradeoff between enzyme and metabolite efficiency maintains metabolic homeostasis upon perturbations in enzyme capacity. *Mol. Syst. Biol.*, 6(356):356.

Kümmel, A. (2008). *Integrating Thermodynamics-based Modeling and Quantitative Experimental Data for Studying Microbial Metabolism.* PhD thesis, ETH Zürich.
