# Supplementary material for: System-Level Insights into Yeast Metabolism by Thermodynamic Analysis of Elementary Flux Modes
Source: PLoS Comput Biol. 2012 Mar 1;8(3):e1002415. doi: 10.1371/journal.pcbi.1002415 (PMC3296127; doi:10.1371/journal.pcbi.1002415)
Supplement: Text S1 — Mathematic proof showing that a flux distribution containing an infeasible EFM is always infeasible. (PDF) [file pcbi.1002415.s004.pdf]

Text S1. Feasibility of Elementary Modes

In this supplement a detailed proof is given showing that a flux distribution containing an infeasible EFM is always infeasible.

Methods

The flux direction for a chemical reaction is related to Gibbs free energy as follows:

$$\begin{aligned} \Delta_r G_j < 0 &\implies v_j > 0 \\ \Delta_r G_j > 0 &\implies v_j < 0 \end{aligned} \tag{1}$$

Here, $\Delta_r G_j$ denotes Gibbs free energy for a reaction j, and v_j is the flux through this reaction. The free energy of a reaction can be calculated from Gibbs energies of formation of the participating reactants ($\Delta_f G$):

$$\boldsymbol{\Delta_r G} = \boldsymbol{S}\,\boldsymbol{\Delta_f G} \tag{2}$$

Note that $\boldsymbol{\Delta_r G}$ and $\boldsymbol{\Delta_f G}$ are vectors of length r and m for a metabolic network with stoichiometric matrix $\boldsymbol{S} \in \mathbb{R}^{m \times r}$ containing m metabolites and r reactions. Gibbs energy of formation for metabolites can be derived from its standard Gibbs energy and the thermodynamic activity. The *network-embedded thermodynamic (NET) analysis* applied here uses measured metabolite concentrations to derive energy of formation for each metabolite (1). Allowing for a certain tolerance due to measurement errors, Gibbs formation energies are constrained to an interval:

$$\boldsymbol{\Delta_f G}_{min} \leq \boldsymbol{\Delta_f G} \leq \boldsymbol{\Delta_f G}_{max} \tag{3}$$

Given a flux vector $\boldsymbol{v}$, NET analysis performs a feasibility analysis, adding additional flux dependent constraints for every non-zero flux value v_j, restricting Gibbs free energy for reaction j according to eq. (1). This yields patterns of reaction directionalities that are not feasible, even if all individual reaction directions are very well possible.

Let us assume for the moment that we have a tool that decides whether a certain flux vector is feasible or not, based on the direction pattern of the flux vector. If we apply the tool to special vectors like elementary modes (EMs), it would be desirable that we could then draw feasibility conclusions also for other flux vectors, for instance for those derived from elementary modes. Ideally, we would like to remove infeasible EMs and shrink the flux cone accordingly. But is this really what we want, i.e. can we still

generate all feasible flux vectors from the remaining feasible EMs? The answer is yes, under certain conditions, and to work towards a proof, we have to concretize some definitions.

Definition 1. *The* flux pattern $\phi(\boldsymbol{v})$ *of a flux vector* $\boldsymbol{v}^{1\times r}$ *are the indices* $j \in \{1,\dots,r\}$ *associated with nonzero fluxes, multiplied with the signum of the flux value. More formally,*

$$\phi(\boldsymbol{v}) = \{\, j \cdot \mathrm{sgn}(v_j) \mid v_j \neq 0\,\} \quad \text{where } \mathrm{sgn}(v_j) = \begin{cases} -1 & \text{if } v_j < 0 \\ 0 & \text{if } v_j = 0 \\ +1 & \text{if } v_j > 0 \end{cases} \tag{4}$$

Note that irreversible reactions can be seen as special case for feasibility conditions, preventing a flux direction of a single reaction. Hence, irreversibility constraints classify flux patterns of size one as infeasible. We can apply conditions for such simple infeasibilities *a priori* to the computation, usually stated as non-negativity constraints for the corresponding variables. More complex conditions are applied *after* the computation of the elementary modes. For those conditions, we need a formal definition of *feasibility classification*:

Definition 2. *A function* $f(\boldsymbol{v}) : \mathbb{R}^d \to [0,1]$ *is called* feasibility classifier *for flux vectors* $\boldsymbol{v}^d$ *if for any flux vector* $\boldsymbol{v}'$ *with* $\phi(\boldsymbol{v}') \supseteq \phi(\boldsymbol{v})$*, infeasibility (*$f = 0$*) of* $\boldsymbol{v}$ *implies infeasibility of* $\boldsymbol{v}'$*:*

$$\phi(\boldsymbol{v}') \supseteq \phi(\boldsymbol{v}) \;,\; f(\boldsymbol{v}) = 0 \quad \Longrightarrow \quad f(\boldsymbol{v}') = 0 \tag{5}$$

The NET analysis mentioned above is an example for a feasibility classifier, but the following is true for *any* classifier conforming with Def. 2: infeasible elementary modes can be removed from the generating matrix without losing feasible flux vectors. Equivalently, we can compose any feasible flux vector from feasible elementary modes, as stated formally by the following theorem:

Theorem 1. *Let* f *be an feasibility classifier according to Def. 2, and let* $\mathcal{P}$ *be a flux cone generated by* n *elementary modes* $\boldsymbol{e}_j$ *(see Def. 2.39 and 2.40 in (2)). Let us furthermore denote by* F *be the set of indices associated with feasible EMs, i.e.* $F = \{\, j \mid j \in \{1,\dots,n\} \;,\; f(\boldsymbol{e}_j) = 1\}$*. Then, any feasible flux vector* $\boldsymbol{v} \in \mathcal{P}$ *can be composed solely from feasible elementary modes* $\boldsymbol{e}_k$ *with* $k \in F$*. More formally, we have:*

$$\boldsymbol{v} \in \mathcal{P}, f(\boldsymbol{v}) = 1 \quad \Longrightarrow \quad \forall k \in F, \exists\, \lambda_k \geq 0 \quad \textit{such that} \quad \boldsymbol{v} = \sum_{k \in F} \lambda_k \boldsymbol{e}_k \tag{6}$$

Proof

Let us first establish an important relationship between flux patterns (Def. 1) and zero sets (Def. 2.28, eq. (2.39) in (2)) associated with flux vectors:

Proposition 1. *Let $\boldsymbol{v}_1$ and $\boldsymbol{v}_2$ be flux vectors in $\mathbb{R}^r$ with associated flux patterns $\phi_1 := \phi(\boldsymbol{v}_1)$ and $\phi_2 := \phi(\boldsymbol{v}_2)$. Let $\boldsymbol{v}'_1$ and $\boldsymbol{v}'_2$ be the same vectors projected to the augmented dimensionality space of the EM cone as implied by Def. 2.39 in (2), and $\zeta_1 := \zeta(\boldsymbol{v}'_1)$ and $\zeta_2 := \zeta(\boldsymbol{v}'_2)$ be the corresponding zero sets given by eq. (2.39) in (2). Then, the following holds:*

$$\phi_1 \supseteq \phi_2 \Longleftrightarrow \overline{\zeta_1} \supseteq \overline{\zeta_2} \tag{7}$$

$$\Longleftrightarrow \zeta_2 \supseteq \zeta_1 \tag{8}$$

$$\phi_1 \not\supseteq \phi_2 \Longleftrightarrow \overline{\zeta_1} \not\supseteq \overline{\zeta_2} \tag{9}$$

$$\Longleftrightarrow \zeta_2 \not\supseteq \zeta_1 \tag{10}$$

Proof. Similar to the flux pattern (ϕ), the complement of the zero set ($\overline{\zeta}$) contains indices associated with nonzero flux values. Let us consider any reaction indexed by i. If i is irreversible, we have a one-to-one mapping from ϕ to $\overline{\zeta}$, i.e. both sets contain or do not contain i. If i is reversible and the associated flux vector carries a positive flux value at i, both sets contain i. For a negative flux value, ϕ contains $-i$ and $\overline{\zeta}$ contains $i + r$. For zero flux, neither of the sets contains any of the elements and we have a one-to-one correspondence between the elements in ϕ and $\overline{\zeta}$. From this, eq. (7) and (9) follow immediately. The remaining equivalences are simple set contrapositions. □

Proposition 2. *Let $\boldsymbol{v} = \boldsymbol{q} + \lambda_i \boldsymbol{e}_i$ be a feasible ray composed of a (not necessarily feasible) ray $\boldsymbol{q}$ and an infeasible elementary part $\lambda_i \boldsymbol{e}_i$. Then*

$$\exists \boldsymbol{e} \not\simeq \boldsymbol{e}_i \, : \, \phi(\boldsymbol{e}) \subseteq \phi(\boldsymbol{v}) \tag{11}$$

Proof. At least one flux value of a reversible reaction in $\boldsymbol{e}_i$ has been cancelled out or reverted in $\boldsymbol{v}$, otherwise, $\phi(\boldsymbol{v}) \supseteq \phi(\boldsymbol{e}_i)$, and $\boldsymbol{v}$ would be infeasible according to eq. (5). Due to eq. (10), also $\zeta(\boldsymbol{e}'_i) \not\supseteq \zeta(\boldsymbol{v}')$ holds for the projections to the EM cone (denoted by $'$). Note that elementary modes are not minimal, hence $\boldsymbol{v}$ can be an EM and still composite, and we have

$$\text{either} \qquad \boldsymbol{v} \text{ is an EM} \tag{12}$$

$$\text{or} \qquad \exists \boldsymbol{e} \not\simeq \boldsymbol{e}_i \, : \, \phi(\boldsymbol{e}) \subset \phi(\boldsymbol{v}) \tag{13}$$

This follows from the combinatorial test for extreme rays (Lemma 2.4 in (2)): since $\zeta(\boldsymbol{e}'_i)$ is not a superset of $\zeta(\boldsymbol{v}')$, either another EM $\boldsymbol{e}$ distinct from $\boldsymbol{e}_i$ must exist with

$\zeta(\boldsymbol{e}') \supset \zeta(\boldsymbol{v}')$ and eq. (13) holds, or $\boldsymbol{v}$ is itself an EM, as stated by eq. (12). Eq. (11) is clear if (13) holds. On the other hand, if $\boldsymbol{v}$ is an EM and since $\boldsymbol{v} \not\simeq \boldsymbol{e}_i$, we can set $\boldsymbol{e} = \boldsymbol{v}$ with $\phi(\boldsymbol{e}) = \phi(\boldsymbol{v}) \subseteq \phi(\boldsymbol{v})$. □

Proposition 3. *Let $\boldsymbol{v} = \boldsymbol{q} + \lambda_i \boldsymbol{e}_i$ be a feasible ray composed of a (not necessarily feasible) ray $\boldsymbol{q}$ and an infeasible elementary part $\lambda_i \boldsymbol{e}_i$, and $\boldsymbol{v}$ itself is not an EM, i.e. eq.* (13) *holds. Then*

$$\exists \boldsymbol{e}_1 \not\simeq \boldsymbol{e}_i, \boldsymbol{e} \,:\, \phi(\boldsymbol{e}_1) \subset \phi(\boldsymbol{v}) \tag{14}$$

Proof. Let us introduce a new vector $\boldsymbol{v}_1 = \boldsymbol{v} - \lambda \boldsymbol{e}$. If we choose a minimal $\lambda = \min(\frac{r_j}{e_j})$ from all j with $e_j \neq 0$, at least one flux value of $\boldsymbol{v}$ and $\boldsymbol{e}$ is cancelled in $\boldsymbol{v}_1$. Note that $\lambda > 0$ since $\phi(\boldsymbol{v}) \supset \phi(\boldsymbol{e})$, i.e. all flux value pairs (r_j, e_j) have equal sign for $e_j \neq 0$, and $\boldsymbol{v}_1$ has no inverted sign compared to $\boldsymbol{v}$ due to the minimal choice of λ. Hence, $\phi(\boldsymbol{v}_1) \subset \phi(\boldsymbol{v})$, and consequently, all non-negativity constraints still hold and $\boldsymbol{v}_1$ is a ray of the flux cone.

Furthermore, we know that $\phi(\boldsymbol{v}) \not\supseteq \phi(\boldsymbol{e}_i)$, hence clearly also $\phi(\boldsymbol{v}_1) \not\supseteq \phi(\boldsymbol{e}_i)$, and since we have cancelled out at least one value of $\boldsymbol{e}$, also $\phi(\boldsymbol{v}_1) \not\supseteq \phi(\boldsymbol{e})$. Then,

$$\text{either} \quad \boldsymbol{v}_1 \text{ is an EM} \tag{15}$$

$$\text{or} \quad \exists \boldsymbol{e}_1 \not\simeq \boldsymbol{e}_i, \boldsymbol{e} \,:\, \phi(\boldsymbol{e}_1) \subset \phi(\boldsymbol{v}_1) \tag{16}$$

The reasoning is the same as for (12, 13): either $\boldsymbol{v}_1$ is an EM, or its elementarity is disproved by existence of $\boldsymbol{e}_1$. Since $\phi(\boldsymbol{v}_1) \subset \phi(\boldsymbol{v})$, (14) follows. □

Lemma 1 (Elimination Lemma for Infeasible Elementary Terms)**.** *Let $\boldsymbol{v} = \boldsymbol{q} + \lambda_i \boldsymbol{e}_i$ be a feasible ray composed of a (not necessarily feasible) ray $\boldsymbol{q}$ and an infeasible elementary part $\lambda_i \boldsymbol{e}_i$. Then, there exists some $\boldsymbol{\lambda} \geq \boldsymbol{0}$ such that $\boldsymbol{v} = \sum_{j=1}^{n} \lambda_j \boldsymbol{e}_j$ with $\lambda_j = 0$ if $j = i$, i.e. $\boldsymbol{v}$ can be decomposed into elementary modes without using $\boldsymbol{e}_i$.*

Proof. Using Prop. 2, either $\boldsymbol{v} \not\simeq \boldsymbol{e}_i$ is an EM according to (12) and we're done, or we can apply Prop. 3 and $\boldsymbol{v} = \boldsymbol{v}_1 + \lambda \boldsymbol{e}$. If $\boldsymbol{v}_1$ is an EM (eq. 15), we're done since $\boldsymbol{e}, \boldsymbol{v}_1 \not\simeq \boldsymbol{e}_i$. Otherwise, we reapply the decomposition technique used in Prop. 3 for $\boldsymbol{v}_1$ and $\boldsymbol{e}_1$, i.e. we find a ray $\boldsymbol{v}_2$ such that $\boldsymbol{v}_1 = \boldsymbol{v}_2 + \lambda_1 \boldsymbol{e}_1$.

We continue with the decomposition technique $\boldsymbol{v}_k = \boldsymbol{v}_{k+1} + \lambda_k \boldsymbol{e}_k$ until $\boldsymbol{v}_{k+1}$ is an EM. Note that the newly found elementary modes $\boldsymbol{e}_k \not\simeq \boldsymbol{e}_i$, nor are they equivalent to previously found elementary modes, since $\phi(\boldsymbol{v}) \not\supseteq \phi(\boldsymbol{e}_i)$, and $\phi(\boldsymbol{v}_{k+1}) \subset \phi(\boldsymbol{v}_k) \subset \ldots \subset \phi(\boldsymbol{v}_1) \subset \phi(\boldsymbol{v})$. We can thus rewrite $\boldsymbol{v}$ without using $\boldsymbol{e}_i$, namely $\boldsymbol{v} = \lambda \boldsymbol{e} + \lambda_1 \boldsymbol{e}_1 + \ldots + \lambda_k \boldsymbol{e}_k + \lambda_{k+1} \boldsymbol{e}_{k+1}$ stopping at the elementary $\boldsymbol{v}_{k+1} \simeq \boldsymbol{e}_{k+1}$. □

Decomposition technique and dependencies of flux patterns are visualized in Fig. 1.

Now, we are ready to prove Theorem 1.

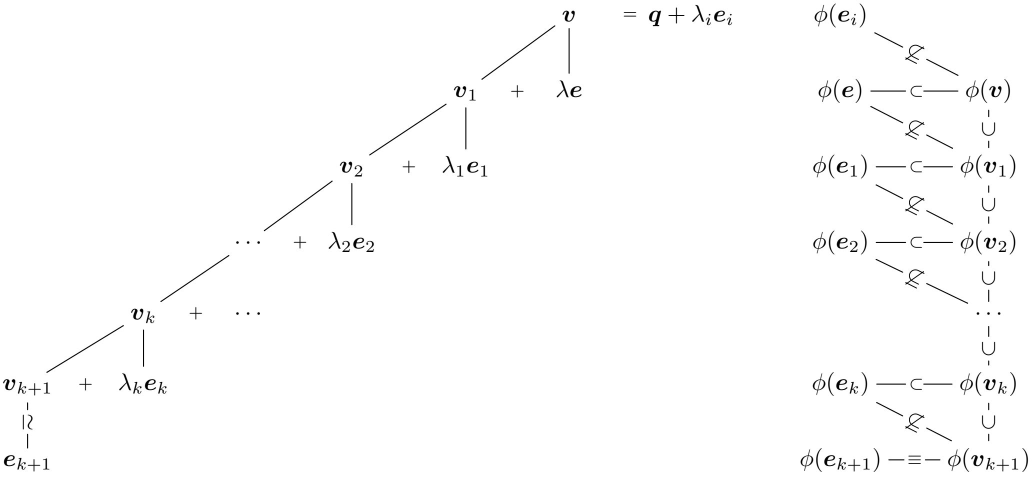

Figure 1: Elimination of infeasible elementary term $\lambda_i \boldsymbol{e}_i$: decomposition of $\boldsymbol{v}$ into elementary modes $\boldsymbol{e}, \boldsymbol{e}_1, \ldots, \boldsymbol{e}_{k+1}$ (left) and depencencies of flux patterns (right).

Proof. Assume that some $\boldsymbol{e}_k$ in eq. (6) is infeasible, and let us denoted it by $\boldsymbol{e}_i$. Using Lemma 1, we can eliminate $\boldsymbol{e}_i$ for every feasible ray $\boldsymbol{v}$ of the flux cone, i.e. we find some $\lambda_k \geq 0$ such that

$$\boldsymbol{v} = \sum_{k \in \{1,\ldots,r\} \setminus \{i\}} \lambda_k \boldsymbol{e}_k \tag{17}$$

Since we can still generate all feasible EMs without $\boldsymbol{e}_i$, we can remove it and consider the reduced flux cone generated by $(n-1)$ elementary modes $[\boldsymbol{e}_k], k \in \{1, \ldots, r\} \setminus \{i\}$. Continuing with the reduced flux cone, we eliminate another infeasible elementary term until no infeasible terms are left. □

References

1. Anne Kümmel, Sven Panke, and Matthias Heinemann. Putative regulatory sites unraveled by network-embedded thermodynamic analysis of metabolome data. *Molecular Systems Biology*, 2:2006.0034, 2006.

2. Marco Terzer. *Large scale methods to enumerate extreme rays and elementary modes.* PhD thesis, ETH Zürich, 2009. URL http://dx.doi.org/10.3929/ethz-a-005945733.
