# Supplementary material for: System-Level Insights into Yeast Metabolism by Thermodynamic Analysis of Elementary Flux Modes
Source: PLoS Comput Biol. 2012 Mar 1;8(3):e1002415. doi: 10.1371/journal.pcbi.1002415 (PMC3296127; doi:10.1371/journal.pcbi.1002415)
Supplement: Text S2 — Additional results regarding flux balance analysis showing the obtained patterns correspond with known operating principles. (PDF) [file pcbi.1002415.s005.pdf]

Text S2. Additional results

Patterns correspond with known operating principles

To determine whether the patterns that we find (Fig. 5 in main text) correspond with current knowledge on metabolic operating principles in yeast we applied a flux balance analysis (FBA) approach to determine intracellular fluxes. Previous work suggests that for specific conditions, specific optimization functions give the most accurate prediction of intracellular fluxes (Schütz et al., 2007; Schütz, 2009). It was identified that for *E. coli* growing in batch conditions maximization of the ratio of ATP overproduction over the sum of squared fluxes gives the most accurate flux prediction (Schütz et al., 2007). For *E. coli* growing in nutrient limited conditions, maximization of ATP overproduction gave the most accurate predictions.

We performed FBA with three objective functions, (1) maximization of biomass production, (2) maximization of ATP overproduction and (3) maximization of the ratio of ATP overproduction over the sum of squared fluxes. The physiological data as described in Supplement 3 was used to constrain the exchange fluxes. In Tab. 1 we show the reaction activities obtained from FBA simulations. In the reaction activities we can see that pattern 1 (see Fig. 5 in main text) occurs in the flux solution obtained by maximizing the biomass production, and patterns 2, 3, 5 and 6 occur in the flux solution obtained by maximizing ATP overproduction. No patterns occur in the solution obtained from the maximization of the ratio of ATP production over the sum of squared fluxes. This corresponds well with the earlier findings that the maximization of ATP overproduction describes the fluxes of *E. coli* under nutrient limited conditions (e.g. chemostat cultivation) and the maximization of ATP production over the sum of squared fluxes describes the fluxes under nutrients in excess. This shows that the infeasibility patterns we find are corresponding with previous findings on metabolic operations in the cell.

References

Schütz, R. (2009). *Model-driven identification of operating principles in metabolic networks.* PhD thesis, ETH Zürich.

Schütz, R., Kuepfer, L., and Sauer, U. (2007). Systematic evaluation of objective functions for predicting intracellular fluxes in Escherichia coli. *Mol. Syst. Biol.*, 3:119.

Table 1: Reaction directions for FBA solutions. We applied FBA to our model with three objective functions: (1) maximization of biomass production, (2) maximization of ATP overproduction and (3) maximization of the ratio of ATP overproduction over the sum of squared fluxes. A "-1" indicates a backward reaction activity and a "1" a forward reaction activity. It can be seen that pattern 1 occurs in the solution with objective function (1) and patterns 2, 3, 5 and 6 in the solution with objective function (2). In the reaction formulas compartments are indicated between square brackets, where [c] indicates cytosol and [m] indicates the mitochondrion.

**Name**	**Reaction equation and direction in pattern**		**Occurring in pattern**	**Direction in solution**		
				(1)	(2)	(3)
ALCD2m	[m] etoh + nad ⇌ acald + h + nadh	1	2 3 5 6	-1	1	-1
ALCD2x	[c] etoh + nad ⇌ acald + h + nadh	-1	2 3	1	-1	-1
FUM	[c] fum + h2o ⇌ mal-L	1	4 6	1	1	1
GAPD	[c] g3p + nad + pi ⇌ 13dpg + h + nadh	1	1 3 5	1	1	1
MDH	[c] mal-L + nad ⇌ h + nadh + oaa	1	4 6	-1	1	1
MDHm	[m] mal-L + nad ⇌ h + nadh + oaa (rev)	-1	1 4	-1	1	1
PGCD	[c] 3pg + nad → 3php + h + nadh	1	2	1	1	1
THRA	[c] acald + gly ⇌ thr-L	1	5 6	1	1	1
TPI	[c] dhap ⇌ g3p	1	3	1	1	1
ACALDtm	acald[c] ⇌ acald[m]	-1	2 3 5 6	1	-1	1
ETOHtm	etoh[c] ⇌ etoh[m]	1	2 3	-1	1	-1
OAAt2m	(2) h[c] + oaa-2[c] ⇌ (2) h[m] + oaa-2[m]	-1	1 4	-1	1	1
THRt2m	thr-L0[c] ⇌ thr-L0[m]	1	5 6	1	1	1
